# Living in extreme environments: distribution of *Lyciumhumile* (Solanaceae), an endemic halophyte from the Altiplano-Puna region, South America

**DOI:** 10.3897/phytokeys.185.71377

**Published:** 2021-11-08

**Authors:** María Virginia Palchetti, Juan José Cantero, Vanezza Morales-Fierro, Gloria E. Barboza, Andrés Moreira-Muñoz

**Affiliations:** 1 Instituto Multidisciplinario de Biología Vegetal, Consejo Nacional de Investigaciones Científicas y Técnicas, Universidad Nacional de Córdoba, Córdoba, Argentina; 2 Departamento de Ciencias Farmacéuticas, Facultad de Ciencias Químicas, Universidad Nacional de Córdoba, Córdoba, Argentina; 3 Departamento de Biología Agrícola, Facultad de Agronomía y Veterinaria, Universidad Nacional de Río Cuarto, Río Cuarto, Argentina; 4 Museo Nacional de Historia Natural, Área Botánica, Santiago, Chile; 5 Instituto de Geografía, Facultad de Ciencias del Mar y Geografía, Pontificia Universidad Católica de Valparaíso, Valparaíso, Chile

**Keywords:** Andes, Argentina, Bolivia, Chile, conservation status, new record, saline soil, salt-tolerant

## Abstract

Very few Solanaceae species are able to grow in saline soils; one of them is *Lyciumhumile*. This species is endemic to the Altiplano-Puna region (Central Andes, South America) where there are multiple extreme environmental conditions such as hypersaline soils. Here we present an updated description and distribution of *L.humile* including its new record for Bolivia at the edges of “Salar de Uyuni”, the largest salt flat in the world; we discuss its ecological role in saline environments by analyzing soil salinity and cover-abundance values ​​of the studied sites. According to IUCN criteria, we recommend a category of Least Concern for *L.humile*, but the growing development of lithium mining in saline environments of the Altiplano-Puna region may potentially threaten exclusive communities.

## Introduction

*Lycium* L. is the only member of tribe Lycieae (Atropina clade, Solanaceae; Hunziker 1977; Levin et al. 2011; Särkinen et al. 2013) and comprises nearly 90 woody species commonly found in arid, sub-arid and even saline environments (Bernardello 2013). It is a cosmopolitan genus with its greatest diversity occurring in extratropical areas, in southern South America, southern Africa, and southwestern North America (Stiefkens et al. 2020). In South America, the shrub endemic to the Altiplano-Puna region *Lyciumhumile* Phil. preferentially inhabits saline soils with a distribution circumscribed to Argentina and Chile (Bernardello 1986, 2013).

The Altiplano-Puna region of the Central Andes is considered a cold desert, with high elevation (3700 m average elev.), extreme temperatures (which can reach –30 °C) and daily temperature fluctuations, low and irregular precipitation (even < 50 mm per year), high evaporation and UV radiation, and low nutrient availability (Alonso 2013; Malatesta et al. 2016). In addition, this region is characterized by numerous salt flats and shallow saline lakes, called salars, with very high salt concentrations (Alonso and Rojas 2020). The edges of these salars, which have rather wet soils in some periods, support specific plant communities comprising *L.humile* together with other species such as *Distichlishumilis* Phil., *Distichlisspicata* (L.) Greene and *Frankeniatriandra* J.Rémy (Cabrera 1957; Luebert and Gajardo 2000; Tálamo et al. 2010; Carilla et al. 2018). Thus, *L.humile* is one of the few vascular plant species growing at these edges since, even though the water table is near to the surface, plants need to cope with high salinity (> 200 mM NaCl) that promote physiologically dry soils. Halophytes, like *L.humile*, are the only plants able to grow in these environments because they have different mechanisms that enhance root water uptake by decreasing their water potential (Palchetti et al. 2020).

As part of a study of salt tolerance in South American Solanaceae species, we carried out extensive explorations in the Altiplano-Puna region to check the presence and abundance of Solanaceae, in extreme saline environments. Therefore, the aims of this study are: 1) to update the distribution and description of *L.humile*, 2) to document a new record for Bolivia, and 3) to discuss the ecological role of this species in saline environments of the Altiplano-Puna region.

## Methods

### Field trips

Field collections were performed in Argentina, Bolivia and Chile during 2015 to 2019, comprising expeditions carried out in Jujuy, Salta and Catamarca provinces (Argentina), Antofagasta region (Chile) and Potosí department (Bolivia), between 2000 and 4000 m elevation.

### Morphology, vernacular names and uses

Species description was based on *Lycium* monograph (Bernardello 1986) and field observations of 16 populations. Phenology was recorded by observation of plants in the field and herbaria specimens; plant-consuming animals were also annotated. Vernacular names and uses are based on the following authors, Aldunate et al. (1981), Bernardello (1986, 2013), Villagrán and Castro (2003), Philippi (2008), Medina (2012) and Gamboa Fuentes (2014).

### Distribution

Distribution was plotted using QGIS 2.8 (QGIS Development Team 2018), based on data from *Lycium* monograph (Bernardello 1986), revised, digitized or original herbarium specimens (BA, BAA, CONC, CORD, E, EIF, F, K, L, LIL, LP, LPB, MA, MO, SGO, SI; acronyms following Thiers 2021), GBIF.org (2020) database, and data from field trips. Non-georeferenced localities were checked by the authors. No *L.humile* voucher was found in the Bolivian herbaria LPB and USZ. Analyzed data are available as supplementary information (see Suppl. material 1: Palchetti et al. SF1).

**Figure 1.**
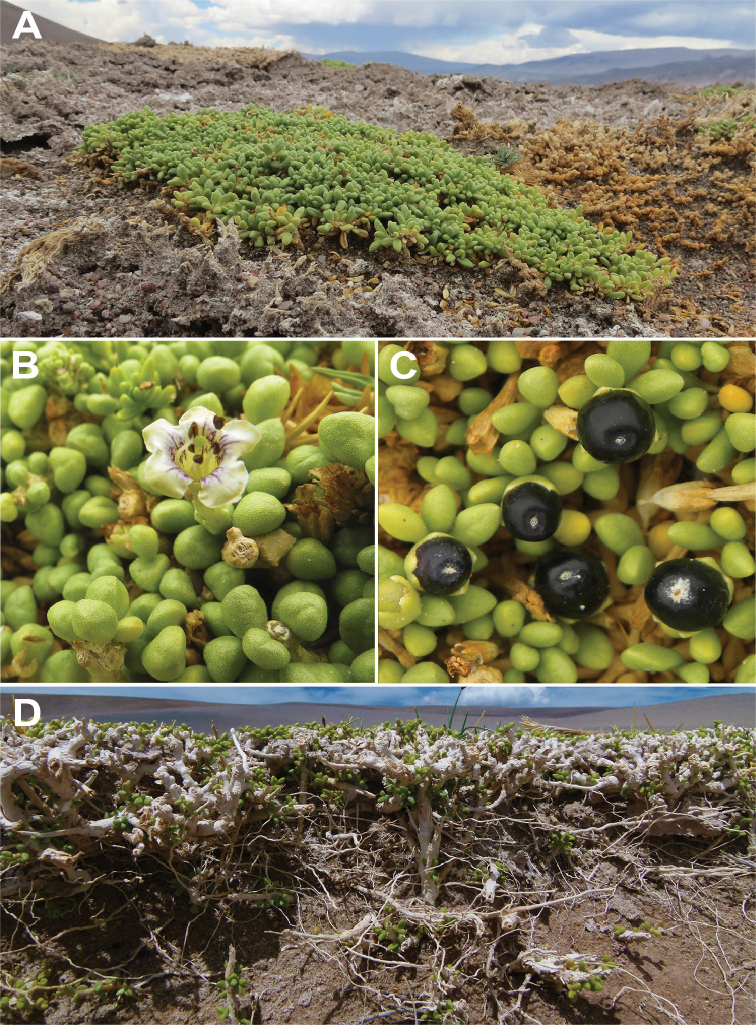
*Lyciumhumile***A** overview of a plant in its habitat **B** flower **C** fruits **D** plant architecture.

### Habitat and ecology

Local abundance of *L.humile* and different edaphic variables (pH, electric conductivity [EC], Na^+^, Cl–, Ca^2+^ and Mg^2+^) were evaluated in seven sites of Argentina, where geographic coordinates were recorded; voucher specimens were collected and deposited at CORD herbarium. Local abundance was calculated as estimated *L.humile* cover percentage in thirty 10 m × 10 m plots at each site. Fifteen random soil sub-samples at 0–20 cm depth were taken from each site and pooled as a composite sample for saturation extract analysis (Richards 1954) performed by LabSA, FCA-UNC (Córdoba, Argentina).

**Figure 2.**
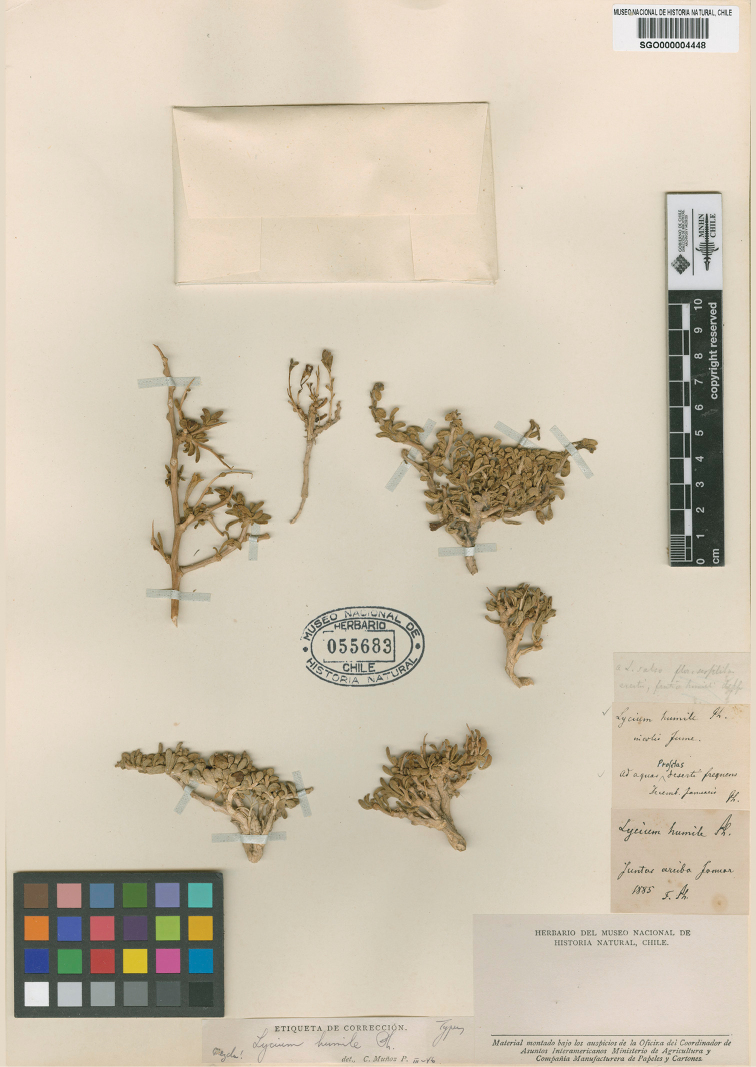
Lectotype of *Lyciumhumile* Phil. (SGO 055683). Digital image by courtesy of the Museo Nacional de Historia Natural.

**Figure 3.**
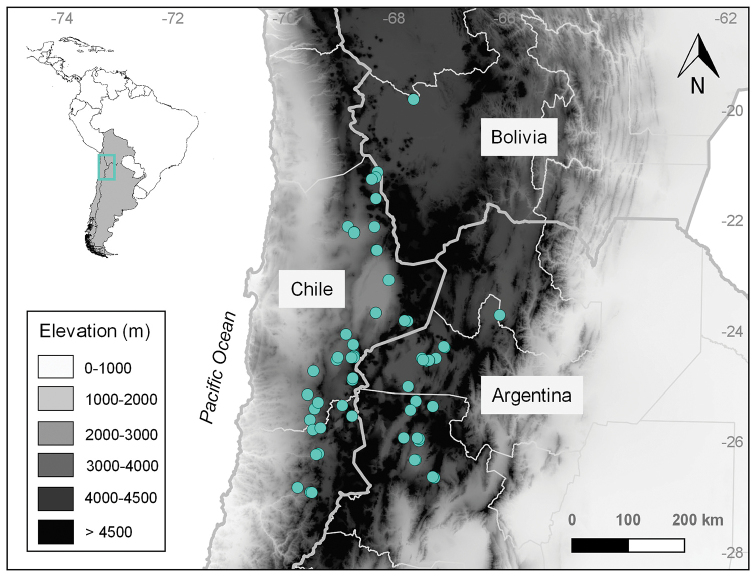
Distribution map of *Lyciumhumile* (light-blue circles) in the Altiplano-Puna region.

### Conservation status

The conservation status was assessed applying the IUCN (2019) criteria. The extent of occurrence and area of occupancy were calculated using a convex hull in QGIS 2.8 (QGIS Development Team 2018) and the Geospatial Conservation Assessment Tool, GeoCAT (Bachman et al. 2011), respectively.

## Taxonomic treatment

### Lycium humile

Taxon classificationPlantaeSolanalesSolanaceae

Phil., Fl. Atacam. 43. 1860.

48820BF0-FA2A-585C-9BB5-AEA653AE261C

Fig. 1

#### Type.

Chile. [Antofagasta: Province of Antofagasta], Ad aquas [Profetas], December [1853]–January [1854], *R.A. Philippi s.n.*, pro parte (lectotype, inadvertently designated as ‘type’ by Muñoz Pizarro 1960, pg. 116: SGO! [SGO000004448, acc. # 055683; Fig. 2], isolectotype: W n.v. [cited as isosyntype by Bernardello (1986)].

#### Description.

Dwarf shrubs, prostrate or ascending, up to 20 cm high, often forming dense and extensive mats, over 5 m. Subterranean organs well-developed with tangled and woody roots and rhizomes. Stems grayish-yellow, unarmed, glabrous, much branched, with slightly arched aerial branches and some stoloniferous branches. Leaves alternate or fasciculate, succulent, obovate or spatulate, light green, 2–16 mm long, 1–4 mm wide, glabrous or with occasionally glandular trichomes, sessile. Flowers 5-merous (rarely 4- or 6-merous), solitary, perfect; calyx tubular, zygomorphic, glabrous, bilabiate or irregularly toothed, the tube 3–5 mm long, the lobes sub-triangular, 1–2 mm long, ciliate at the margins; corolla white, sometimes with purple lines within, narrowly infundibuliform to tubular, barely zygomorphic, glabrous outside, the tube 12–15.5 mm long, 3–3.5 mm wide, glabrescent near the ​​insertion of the stamens inside, the lobes 2.5–3.5 mm long, 2.5–4 mm wide, ovate, with sparse cilia on the edge; stamens inserted at 2/3 from the base, at different levels, filaments unequal in length, some exserted, others included or barely exserted, with few simple hairs at their bases; ovary with prominent red-orange nectary at the base, style exserted or scarcely exserted. Berry subglobose, ca. 7–8 mm in diameter, blackish; seeds irregular, polyhedral, pale brown, up to 25 per fruit, the episperm smooth, without marked cells.

#### Phenology.

Flowering late September-March; fruiting late December-April.

#### Vernacular names and uses.

Bálsamo finito, ch’ampita (Villagrán and Castro 2003); jume (Philippi 2008; Medina 2012); sacha uva or sachauva (Villagrán and Castro 2003; Bernardello 2013); tomatillo (Villagrán and Castro 2003; Medina 2012); uvilla (Villagrán and Castro 2003); walcha (Aldunate et al. 1981; Villagrán and Castro 2003); waycha (Villagrán and Castro 2003; Medina 2012), wicha (Villagrán and Castro 2003; Medina 2012; Gamboa Fuentes 2014). This species has been reported as fodder (Aldunate et al. 1981; Villagrán and Castro 2003; Gamboa Fuentes 2014) and probably has medicinal uses associated with rituals to remove evils (Villagrán and Castro 2003; Medina 2012). Fruits have been reported as edible (Philippi 2008) and may have tinctorial properties (Bernardello 1986; Villagrán and Castro 2003; Medina 2012; Gamboa Fuentes 2014).

#### Distribution.

*Lyciumhumile* is distributed in the Andean region, southern South America (Argentina, Bolivia and Chile), at 2300–4100 m elevation (Fig. 3). In northwestern Argentina, it inhabits sites at 3000–4000 m elev. in the provinces of Catamarca (Antofagasta de la Sierra and Belén departments) and Salta (Los Andes department), and there is also a specimen collected in Jujuy province, Tumbaya department (Ancibor and Ruthsatz 65; BAA). In northeastern Chile, it occurs throughout the species’ elevation range, in the regions of Antofagasta (Antofagasta and El Loa provinces) and Atacama (Copiapó and Chañaral provinces). In southwestern Bolivia, it grows in Potosí department, at the edges of Salar de Uyuni at ca. 3600 m elev. Previous publications (Bernardello 1986; Rodriguez et al. 2018; Zuloaga et al. 2019) have cited the species in the Chilean region of Tarapacá, however after studying herbarium material from Chile we consider its presence on this administrative region as doubtful (see discussion).

#### Habitat and ecology.

**Figure 4.**
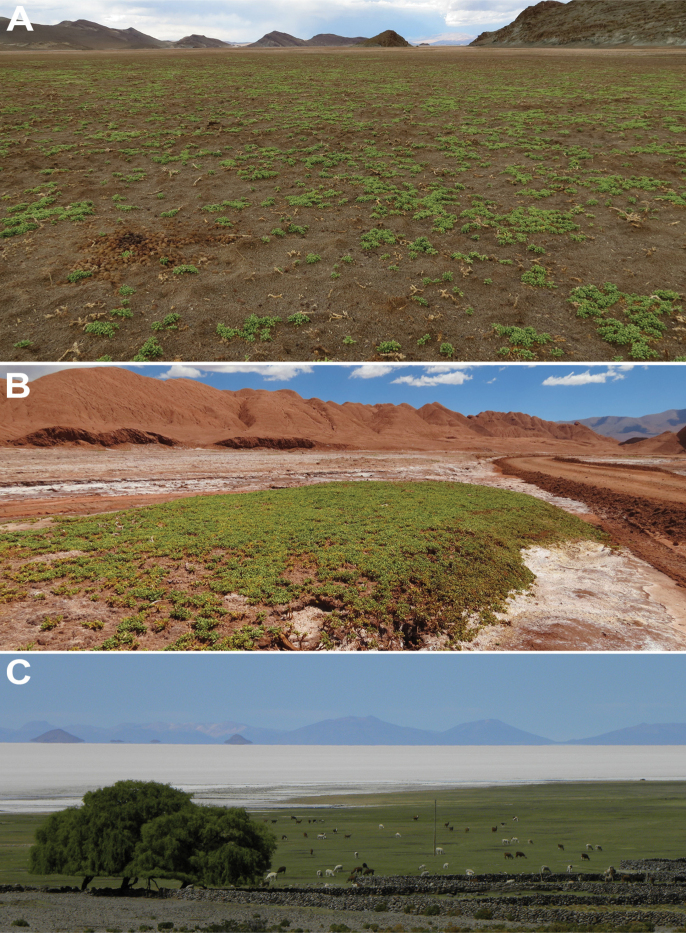
Saline environments of the Altiplano-Puna region (South America) in which *Lyciumhumile* grows **A** Salar del Hombre Muerto (Catamarca, Argentina) **B** Los Colorados (Salta, Argentina) **C** Salar de Uyuni (Potosí, Bolivia).

The species preferentially grows in saline clay soils, and less frequently in sandy soils. It is commonly found in saline mudflats of salars (Fig. 4). In the analyzed sites of the Altiplano-Puna region where *L.humile* grows, soils showed very high salinity reaching high EC (~300 dS/m) and Na^+^ (~30 g/L) values, and low vegetation cover, with an average bare soil of 55 (SD 20.9) %. *Lyciumhumile* showed an average cover percentage of 22.9 (SD 11) % with a maximum value of ~40% in Salar de Antofalla (Catamarca, Argentina, Table 1). The species occurs in plant communities with low species richness, along with Amaranthaceae, *Nitrophilaaustralis* Chodat & Wilczek and *Salicorniapulvinata* R.E.Fr.; Asteraceae, *Baccharisacaulis* (Wedd. ex R.E.Fr.) Cabrera; Frankeniaceae, *Frankeniatriandra*; Juncaginaceae, *Triglochinconcinna* Burtt Davy; and Poaceae, *Distichlishumilis* and *D.spicata*.

#### Conservation status.

According to the IUCN criteria (IUCN 2019), a category of Least Concern (LC; B, C and D criteria) is recommended for *Lyciumhumile*, based on its extent of occurrence of 190,477 km^2^, area of occupancy of more than 2,000 km^2^, and large population size with more than 10,000 mature individuals observed. Large mining operations in the Andes may produce a continuing decline of area, extent and/or quality of habitat (Schiaffini 2013; Liu et al. 2019) which could adversely affect some subpopulations located at specific sites of the salars. But considering its widespread occurrence and healthy populations found in several locations within protected areas in Argentina (Laguna Blanca Biosphere Reserve, Lagunas Altoandinas y Puneñas de Catamarca Ramsar Site and Los Andes Provincial Reserve) and Chile (Llullaillaco National Park), this activity may not represent a current threat to this species at regional scale.

**Table 1.** Edaphic variables of saturated paste extract, bare soil and soil covered by *L.humile* for each study site. Specimen vouchers were deposited at CORD.

**Site**	**Geographical coordinates**	**Specimen voucher**	**Edaphic variables**						**Bare soil (%)**	**Soil covered by *L.humile* (%)**
			**Cl–(mg/L)**	**Ca^2+^ (mg/L)**	**Mg^2+^ (mg/L)**	**Na^+^ (mg/L)**	**pH**	**EC (dS/m)**		
Laguna Pasto Ventura	26°44.0133'S; 67°9.4433'W	Barboza G.E. et al. 4725	1134.0	48.0	14.0	2144.1	7.4	99.2	15.6	16.9
Carachi Pampa	26°26.0633'S; 67°29.38'W	Barboza G.E. et al. 4304	14512.4	1500.0	1518.8	12000.0	9.8	112.5	70.6	17.7
Salar de Antofalla	25°31.93'S; 67°34.855'W	Barboza G.E. et al. 4313	23181.5	480.0	60.8	14875.0	8.7	160.0	52.8	39.2
Salar del Hombre Muerto	25°27.8017'S; 67°10.37'W	Barboza G.E. et al. 4309b	31311.0	1800.0	741.2	17125.0	8.3	182.5	55.5	34.4
Salar Tolar Grande	24°35.575'S; 67°23.4783'W	Barboza G.E. et al. 4749	3200.0	25.0	4.0	20281.7	7.9	203.3	61.5	12.8
Los Colorados	24°35.4217'S; 67°8.2083'W	Barboza G.E. et al. 4347	45191.5	440.0	2940.3	25375.0	8.5	290.0	82.0	28.1
Salar del Diablo	24°37.8667'S; 67°15.7667'W	Barboza G.E. et al. 4349	46576.0	580.0	437.4	29375.0	8.0	290.0	47.2	11.2
**Total average**			23586.6	696.1	816.6	17310.8	8.4	191.1	55.0	22.9
**Standard deviation (SD)**			18516.6	690.5	1082.3	8977.8	0.7	76.8	20.9	11.0

#### New country record.

Bolivia. Potosí: Dept. Daniel Campos, Uyuni, entrando a Coqueza por el propio salar de Uyuni; 9°54.2333'S; 67°37.3667'W; 3665 m elev.; 13 Dec. 2017; G.E. Barboza 4868 (CORD00086059; LPB).

#### Taxonomic note.

R.A. Philippi (1860) described *L.humile* based on his plant collections from his trip to the Atacama Desert during the summer of 1853–1854. The protologue mentioned three localities in Antofagasta, Chile: “primum prope Cachiyuyal 25°22' lat. m. 4000 p.s.m. legi, deinde ad aquam Profetas dictam 24°45' lat. m., 9000 p.s.m., in valle Chaco 25°15' lat. m., 8500 p.s.m.”. Of these three syntypes, Muñoz Pizarro (1960) found a single sheet at SGO (acc. # 055683; Fig. 2) that matches the species and the protologue, being designated by him as “type” of *L.humile*. We consider his citation as an inadvertent lectotypification (see article 7.11 in Turland et al. 2018).

The sheet SGO 055683 bears two labels with different localities, collectors and dates. One label reads “*Lyciumhumile* Ph., incolis Jume, Ad aquas [Profetas] deserti frequens, Decembri. Januario, Ph.”, it agrees with the protologue. The other label reads “*Lyciumhumile* Ph., Juntas arriba, januar 1885, F. Ph.”, which belongs to a collection done by F. Philippi after the description of the species (1860) and is therefore not original material. All material mounted on this single herbarium sheet belongs to *L.humile* and it is impossible to recognize the branches which were gathered by R.A. Philippi. In fact, the sheet also has a label written by Muñoz Pizarro indicating that there is a mix (‘mezcla!’), which likely refers to mixed material.

After the work of Muñoz Pizarro, Bernardello (2013) selected as the lectotype of *L.humile* the specimen SGO 055684, whose label reads “Encantada, Chaco”. Although the second locality “Chaco” is mentioned in the protologue, this specimen cannot be considered as lectotype because it was collected by K. Reiche (inferred by the handwriting of the collector; Muñoz-Schick et al. 2012) after the publication of the protologue and therefore it is not original material (see article 9.4 in Turland et al. 2018).

## Discussion

*Lyciumhumile* is easily identified by the prostrate or even mat-forming growth habit and very succulent leaves, and during summer, by the numerous white flowers and blackish berries, which also grow almost on the soil surface. In addition, aerial organs represent a smaller part of the total plant architecture than the very well-developed subterranean organs which may help to reduce water loss (Palchetti et al. 2021).

*Lycium* is the Solanaceae genus with the highest number of reported taxa growing in saline environments on a global scale (eHALOPH 2021) within the family. In this study, we document that *L.humile* is widely distributed in the Altiplano-Puna region and grows almost exclusively in saline soils. Despite phytosociological studies done in salars of the Bolivian Altiplano (Navarro 1993), *L.humile* was not previously recorded in this area. Our new record not only constitutes the northernmost point of the species distribution range but also offers a valuable contribution to the global knowledge of the halophytic vegetation, since this species grows at the edges of northern Salar de Uyuni. This is the largest salt flat in the world and has amazingly extreme environmental conditions, such as hypersalinity, intense UV irradiance, high lithium concentration and low precipitation, that promote the development of extreme halophiles (Haferburg et al. 2017; Vargas-Cuentas and Roman-Gonzalez 2017).

Further botanical explorations are encouraged to increase the collection in Bolivia and also, to verify if the species grows in the region of Tarapacá, which would represent its northern limit of distribution in Chile. This is because the only specimens related to this area are probably duplicates (K000586026 and CORD00021076) and were labelled without a precise locality. The label of the specimen at K reads “*Lyciumhumile* Ph. Chili, Com. R.A. Philippi 2/1888. Tarapacá”, while the label of the specimen at CORD states “*Lyciumhumile* Phil. Chile. Prov. Tarapacá: Tarapacá. Leg. R.A. Philippi”. These collections were probably collected by F. Philippi and C. Rahmer, during the expedition to the province of Tarapacá in 1885 and distributed (communicated) by R.A. Philippi in 1888. Several specimens from this trip were distributed to foreign herbaria, only with the name of the species and “Tarapacá”, despite the trip covering the High Andes from Copiapó to Pica, going through Antofagasta de la Sierra (Argentina). As there are no other specimens from Tarapacá region, the presence of the species in this region is doubtful. A similar situation occurs in Jujuy province (Argentina), where the only specimen was collected 50 years ago and, despite our expeditions, we have not found the species in the surrounding area of the specimen collection site or in other saline environments of this province such as Salinas Grandes and Salar de Olaroz.

In the studied sites, *L.humile* was one of the dominant species, reaching covers higher than 35% in sites with high bare soil (> 50%). This evidences its essential role as primary producer in this extreme ecosystem since *L.humile* contributes greatly to the composition and structure of vegetation in saline environments of the Altiplano-Puna region. In fact, *L.humile* is the characteristic species of an association and alliance described in Chile (Luebert and Gajardo 2000). At the same time, the branches and leaves of *L.humile* provide material for the construction of shelters for rodents of the genus *Ctenomys* (Rodentia, Ctenomyidae), and food for lizards of the genus *Liolaemus* (Squamata, Liolaemidae), that feed on the berries, facilitating the dispersal and germination of seeds (Abdala et al. 2021). Other South American animals that feed on *L.humile*, e.g. camelids (such as “vicuña” and “llama”) consume aerial organs while birds (like *Muscisaxicola*; Passeriformes, Tyrannidae) eat the berries (pers. obs.); in this sense seed dispersal by birds has been previously reported (Teillier 2000). Thus, beyond our suggested conservation status category of Least Concern for *L.humile*, saline environments of the Altiplano-Puna region should be considered as priority areas for conservation, since the growing development of mining activities may potentially threaten exclusive plant communities (pers. obs.). Especially with the rise of lithium mining during the last eight years due to the increasing demand for lithium-ion batteries (Eftekhari 2017; Kim et al. 2019), which could affect some *L.humile* populations (e.g. in Antofalla, Catamarca, Argentina and in Salar de Atacama, Antofagasta, Chile). In fact, lithium mining has led to socio-environmental conflicts and even it has been proposed as one of the major causes of local environmental degradation (Schiaffini 2013; Liu et al. 2019).

Halophytes represent fewer than 2% of the Angiosperms (Bromham et al. 2020) and within Solanaceae, barely 1.7% of total species are halophytes (Moray et al. 2015). However, there is a growing interest in salt-tolerant plants, not only because saline environments are major contributors to biodiversity (Flowers and Muscolo 2015), but also because these plants represent a powerful tool to understand salt tolerance, a potential source of salt-responsive genes and promoters (Jha et al. 2019), and an alternative source of food, oil raw material, bioenergy and secondary metabolites (Shamsutdinov et al. 2017; Nikalje et al. 2019). Because of the high economic importance of Solanaceae as food and drug sources (e.g. potato, tomato, eggplant, pepper, tobacco), the understanding of their mechanisms of salinity tolerance are fundamental for genetic improvement. Thus, the key role of *L.humile* in saline environments of the Central Andes and its high salt tolerance make it a potential experimental model to study tolerance to salinity and responses to multiple stresses in Solanaceae.

## Supplementary Material

XML Treatment for Lycium humile

## Supplementary materials

Supplementary material 1 Occurences of *Lyciumhumile* in South America Data type occurences Explanation note Living in extreme environments: distribution of *Lyciumhumile* (Solanaceae), an endemic halophyte of the Altiplano-Puna region, South America. https://binary.pensoft.net/file/607706This dataset is made available under the Open Database License (http://opendatacommons.org/licenses/odbl/1.0/). The Open Database License (ODbL) is a license agreement intended to allow users to freely share, modify, and use this Dataset while maintaining this same freedom for others, provided that the original source and author(s) are credited.María Virginia Palchetti, Juan José Cantero, Vanezza Morales-Fierro, Gloria E. Barboza, Andrés Moreira-Muñoz
