# Supplementary material for: Living in extreme environments: distribution of Lyciumhumile (Solanaceae), an endemic halophyte from the Altiplano-Puna region, South America
Source: PhytoKeys. 2021 Nov 8;185:1–15. doi: 10.3897/phytokeys.185.71377 (PMC8596562; doi:10.3897/phytokeys.185.71377)
Supplement: Supplementary material 1 — Occurences of Lyciumhumile in South America [file phytokeys-185-001-s001.pdf]

Country	Locality	Latitude	Longitude	Elevation (m)	Collector or Author	Date	Herbaria* or Source
Argentina	Catamarca, Antofagasta de la Sierra: A ca. 1 km de Antofagasta de la Sierra, rumbo a Antofalla	-26.03747222	-67.43983333	3410	Barboza *et al.* 4746	08 Feb 2017	CORD
Argentina	Catamarca, Antofagasta de la Sierra: Alrededores de Antofagasta de la Sierra	-26.08779	-67.418424	3340	Guller R. (observation)	22 Oct 2014	GBIF.org**
Argentina	Catamarca, Antofagasta de la Sierra: Antofagasta [Alta]	-26.049048	-67.413115	3373	F. Philippi s.n. (SGO acc. # 042849)	Jan 1885	SGO
Argentina	Catamarca, Antofagasta de la Sierra: Antofagasta de la Sierra, justo en el desvío entre Calalaste y Quebrada del Diablo, rumbo a Antofalla	-26.03486111	-67.44858333	3430	Barboza *et al.* 4747	08 Feb 2017	CORD
Argentina	Catamarca, Antofagasta de la Sierra: Antofalla a Antofagasta de la Sierra. Quebrada del Diablo	-26.037199	-67.692496	3945	Cocucci A.A. 1343	18 Jan 2001	CORD
Argentina	Catamarca, Antofagasta de la Sierra: Borde de Salina de Carachi Pampa	-26.43144444	-67.47052778	3023	Barboza *et al.* 4308	04 Feb 2015	CORD
Argentina	Catamarca, Antofagasta de la Sierra: desde Antofalla a Tolar Grande. Borde Salar de Antofalla	-25.358583	-67.483806	3343	Palchetti M.V. (observation)	09 Feb 2017	pers. obs.
Argentina	Catamarca, Antofagasta de la Sierra: desde Antofalla a Tolar Grande. Borde Salar de Antofalla	-25.368583	-67.484167	3332	Palchetti M.V. (observation)	09 Feb 2017	pers. obs.
Argentina	Catamarca, Antofagasta de la Sierra: Incahuasi, Vega Aguadita	-25.455742	-67.181614	3950	Hueck 503	01 Apr 1950	LIL
Argentina	Catamarca, Antofagasta de la Sierra: Salar de Antofalla	-25.53216667	-67.58091667	3353	Barboza *et al.* 4313	05 Feb 2015	CORD
Argentina	Catamarca, Antofagasta de la Sierra: Salar del Hombre Muerto	-25.46336111	-67.17283333	3978	Barboza *et al.* 4309b	04 Feb 2015	CORD
Argentina	Catamarca, Antofagasta de la Sierra: Salar del Hombre Muerto	-25.457952	-67.17787	3967	Wheeler L.C. (observation)	09 Feb 2019	GBIF.org**
Argentina	Catamarca, Antofagasta de la Sierra: Salar del Hombre Muerto	-25.457243	-67.177375	3968	Wheeler L.C. (observation)	09 Feb 2019	GBIF.org**
Argentina	Catamarca, Antofagasta de la Sierra: Salar del Hombre Muerto, sobre el salar	-25.45722222	-67.17722222	3979	Deanna 384	09 Feb 2019	CORD
Argentina	Catamarca, Antofagasta de la Sierra: Salina de Carachi Pampa	-26.43438889	-67.48966667	3012	Barboza *et al.* 4304	04 Feb 2015	CORD
Argentina	Catamarca, Belén: Laguna Pasto Ventura	-26.752634	-67.125206	3614	Wheeler L.C. (observation)	10 Feb 2019	GBIF.org**
Argentina	Catamarca, Belén: Laguna Pasto Ventura	-26.752946	-67.125287	3612	Wheeler L.C. (observation)	10 Feb 2019	GBIF.org**
Argentina	Catamarca, Belén: Laguna Pasto Ventura, unos 2 km desvío de la RP 43 hacia la izquierda desde Villavil rumbo a Antofagasta de la Sierra	-26.73555556	-67.15738889	3620	Barboza *et al.* 4725	10 Mar 2016	CORD
Argentina	Jujuy, Tumbaya: Camino a San Antonio de Los Cobres, 20 km de Tres Morros	-23.785845	-65.9916	3412	Ancibor & Ruthsatz 65	06 Sep 1971	BAA
Argentina	Salta, Los Andes: a 25 km de Tolar Grande, yendo hacia San Antonio de los Cobres	-24.64311111	-67.29583333	3822	Barboza *et al.* 4751	09 Feb 2017	CORD
Argentina	Salta, Los Andes: Aguas Amargas, camino a Incahuasi "Borde del Salar de Pocitos"	-24.39015	-66.980605	3657	Cabrera 8878	03 Feb 1946	LP
Argentina	Salta, Los Andes: Camino de San Antonio de Los Cobres a Salar de Arizaro, Salar del Diablo	-24.631515	-67.258614	3796	Ancibor & Ruthsatz 67	08 Sep 1971	BAA
Argentina	Salta, Los Andes: Los Colorados, rumbo a Tolar Grande	-24.59036111	-67.13680556	3765	Barboza *et al.* 4347	11 Mar 2015	CORD
Argentina	Salta, Los Andes: Ruta 59, Salar del Diablo, entre Pocitos y Abra de Navarro	-24.63126	-67.25859	3796	Cabrera 31794	16 Feb 1980	SI
Argentina	Salta, Los Andes: Saladillo del Diablo	-24.631515	-67.258614	3796	Cabrera 8774	15 Feb 1945	LP
Argentina	Salta, Los Andes: Salar de Arizaro (extremo sur), viniendo desde Antofalla	-25.11077778	-67.63022222	3507	Barboza *et al.* 4748	08 Feb 2017	CORD
Argentina	Salta, Los Andes: Salar de Pocitos, justo al borde de la salina, alrededores de localidad Pocitos	-24.37605556	-66.99555556	3671	Barboza *et al.* 4752	09 Feb 2017	CORD
Argentina	Salta, Los Andes: Salar del Diablo, rumbo a Tolar Grande	-24.628872	-67.262028	3841	Barboza *et al.* 4349	11 Mar 2015	CORD
Argentina	Salta, Los Andes: Tolar Grande, alrededores del pueblo	-24.59291667	-67.39130556	3512	Barboza *et al.* 4749	09 Feb 2017	CORD
Argentina	Salta, Los Andes: Tolar Grande, Ojos de Mar	-24.62291667	-67.36827778	3524	Barboza *et al.* 4750	09 Feb 2017	CORD
Bolivia	Potosí, Daniel Campos: Uyuni, entrando a Coqueza por el propio salar de Uyuni	-19.903889	-67.622778	3665	Barboza 4868	03 Dec 2017	CORD, LPB
Chile	Antofagasta, Antofagasta: Ad aquas [Profetas]	-24.8572222	-69.34666667	2577	R.A. Philippi s.n. (SGO acc. # 055683)	Dec [1953]-Jan [1954]	SGO
Chile	Antofagasta, Antofagasta: Aguada de los Profetas	-24.857388	-69.347	2800	Muñoz C. 3873A	14 Jan 1944	SGO
Chile	Antofagasta, Antofagasta: Aguada del Carretón	-25.539314	-69.315118	3150	Arancio & Squeo 10191	Dec 1996	CONC
Chile	Antofagasta, Antofagasta: Aguada Escondida	-25.286128	-69.44592	2750	Arancio & Squeo 10486	Feb 1997	CONC
Chile	Antofagasta, Antofagasta: Nuevo Juncal	-25.73989	-69.39269	2650	Arancio & Squeo 10164	Dec 1996	CONC
Chile	Antofagasta, Antofagasta: Parque Nacional Llullaillaco	-24.57225	-68.612444	4042	Luebert 67.1	Jul 1997	EIF
Chile	Antofagasta, Antofagasta: Parque Nacional Llullaillaco, Salar de Punta Negra	-24.648437	-68.928479	2950	Pardo 9	Feb 2001	CONC
Chile	Antofagasta, Antofagasta: Parque Nacional Llullaillaco, Qubrada Zorras	-24.57225	-68.612444	4042	Baines *et al.* 273	02 Dec 2008	E
Chile	Antofagasta, Antofagasta: Quebrada del Chaco	-25.424021	-69.251212	3180	Arancio & Squeo 10306	Dec 1996	CONC
Chile	Antofagasta, Antofagasta: Quebrada Zorrita, Llullaillaco	-24.6	-68.61666667	3900	Ackermann 118	08 Mar 2001	SGO
Chile	Antofagasta, Antofagasta: Salar de Aguas Calientes	-25.009526	-68.636264	3640	Arroyo *et al* . 94106	18 Jan 1994	CONC
Chile	Antofagasta, Antofagasta: Salar de Imilac	-24.18333333	-68.76666667	2978	Arancio 92-322	13 Mar 1992	CONC
Chile	Antofagasta, Antofagasta: Salar de Imilac	-24.1833	-68.7667	2940	Rodríguez & Ruiz 3556	01 May 1997	CONC, MA
Chile	Antofagasta, Antofagasta: Sierra Almeida, Aguada Pajonales	-24.36666667	-68.63333333	3500	Biese 244	07 Dec 1947	SGO
Chile	Antofagasta, Antofagasta: Taltal, Salar Punta Negra	-24.603137	-68.907771	3500	Werdermann 1002	Feb 1926	CONC, E, F

Country	Locality	Latitude	Longitude	Elevation (m)	Collector or Author	Date	Herbaria* or Source
Chile	Antofagasta, Antofagasta: Taltal-interior, aguada de Vaquillas	-25.28608333	-69.44574722	2780	Teillier 8169	10 Jan 2011	CONC
Chile	Antofagasta, Antofagasta: Vega en el Salar de Aguas Calientes	-24.975795	-68.631127	3680	Arancio & Squeo 10479	Feb 1997	CONC
Chile	Antofagasta, Antofagasta: Volcán Llullaillaco	-24.605344	-68.662142	3950	Arroyo *et al* . 94040	14 Jan 1944	CONC
Chile	Antofagasta, El Loa	-22.643865	-68.243758	3158	avocat (observation)	27 May 2019	GBIF.org**
Chile	Antofagasta, El Loa: Borde este Salar de Ascotán	-21.237384	-68.246237	3730	Patricio Medina 3696	08 Dec 2017	EIF
Chile	Antofagasta, El Loa: Calama, Pampa Cere (salar)	-22.23333333	-68.76666667	2652	Teillier & Anabalón 2980	02 Sep 1992	SGO
Chile	Antofagasta, El Loa: Chiu-Chiu	-22.33333333	-68.65	2320	Lira *et al.* 1	28 Apr 1976	SGO
Chile	Antofagasta, El Loa: Chiu-Chiu, en entrada del pueblo, desde Calama rumbo al paso Ollague	-22.34	-68.652222	2526	Barboza 4867	02 Dec 2017	CORD
Chile	Antofagasta, El Loa: Río Salade valley	-22.64622222	-68.23283333	3094	Baines *et al.* 253	01 Dec 2008	CONC
Chile	Antofagasta, El Loa: Ruta 23, de Socaire a Paso Sico, salar de Aguas Calientes	-23.924167	-67.662222	3950	Zuloaga 11168	24 Feb 2009	SI
Chile	Antofagasta, El Loa: Salar de Ascotán	-21.71666667	-68.26666667	3800	Teillier 4220	Feb 1997	CONC, SGO
Chile	Antofagasta, El Loa: Salar de Atacama, Tilopozo, callejón	-23.78333333	-68.23333333	2301	Vidal 16	15 Nov 1992	SGO
Chile	Antofagasta, El Loa: Salar de Atacama, W de Tilopozo	-23.78333333	-68.23333333	2315	Teillier & Torres-Mura 3328	30 Sep 1993	SGO
Chile	Antofagasta, El Loa: Salar de Carcote	-21.331599	-68.278594	3740	Robres s.n. (MO1386261)	09 Dec 1977	MO
Chile	Antofagasta, El Loa: Salar San Martín	-21.36666667	-68.35	3692	Biese 3054	27 Nov 1949	SGO
Chile	Antofagasta, El Loa: San Pedro de Atacama, Socaire, Salar de Aguas Calientes II	-23.91666667	-67.71666667	4119	Teillier 4082	06 Apr 1997	SGO
Chile	Antofagasta, El Loa: Tilopozo, Salar de Atacama	-23.78333333	-68.23333333	2300	Teillier 3363	07 Nov 1994	SGO
Chile	Antofagasta, El Loa: Toconao, San Pedro de Atacama	-23.183041	-68.01047	2474	Kuschel s.n. (SGO acc. # 066217)	17 Feb 1946	SGO
Chile	Antofagasta, El Loa: Vegas de Turi	-22.230404	-68.285565	3000	Villagrán 2914	17 Jan 1981	CONC
Chile	Atacama, Chañaral: Aguada de la Cruz	-25.920112	-69.33983	2920	Arancio & Squeo 10096	Dec 1996	CONC
Chile	Atacama, Chañaral: Encantada, Chaco	-25.88333333	-69.2	3623	[Reiche s.n.] (SGO acc. # 055684)	[Jan 1896]	SGO
Chile	Atacama, Chañaral: In Campamento Pedernales	-26.350248	-69.226564	3200	Zöllner 3855	20 Jan 1970	L
Chile	Atacama, Chañaral: Pedernales, en el lecho seco de un arroyo	-26.368521	-69.27165	3400	Zöllner 789	12 Jan 1966	CONC
Chile	Atacama, Chañaral: Salar de la Azufrera	-25.4685	-68.811942	3500	Latorre *et al* . 194	23 Feb 2001	CONC
Chile	Atacama, Chañaral: Salar de la Isla	-25.653854	-68.631976	3950	Arroyo *et al* . 94151	22 Jan 1994	CONC
Chile	Atacama, Chañaral: Salar de la Isla	-25.658713	-68.632774	3965	Letelier & Reyes 1113	06 Feb 2007	CONC
Chile	Atacama, Copiapó: Juntas arriba	-27.05444444	-69.36194444	no data	F. Philippi s.n. (SGO acc. # 055683)	Jan 1885	SGO
Chile	Atacama, Copiapó: Las Juntas	-27.05444444	-69.36194444	3070	F. Philippi s.n. (SGO acc. # 042846)	Jan 1885	SGO
Chile	Atacama, Copiapó: Quebrada de Paipote. Extremo superior Vegas La Junta	-27.058478	-69.336778	2940	Marticorenta *et al* . 521	06 Jan 1973	CONC
Chile	Atacama, Copiapó: San Andrés	-26.973533	-69.597083	2500	Johnston 4829	02 Nov 1925	BA

*acronyms following Thiers, B. (2021) *Index Herbariorum: a global directory of public herbaria and associated staff* . New York Botanical Garden’s Virtual Herbarium. Available from: http://sweetgum.nybg.org/science/ih (accessed: 11 May 2021).
**GBIF.org (2020) GBIF Occurrence Download [09 November 2020] https://doi.org/10.15468/dl.skp6qc
